# Accuracy of Proximal and Occlusal Contacts of Single Implant Crowns Fabricated Using Different Digital Scan Methods: An In Vitro Study

**DOI:** 10.3390/ma14112843

**Published:** 2021-05-26

**Authors:** Xi Ren, Keunbada Son, Kyu-Bok Lee

**Affiliations:** 1 Department of Prosthodontics, School of Dentistry, Kyungpook National University, Daegu 41940, Korea; renxi321@gmail.com; 2 Department of Prosthodontics, Shanghai YuJia Dental Clinic, 370 Luochuan East Road, Jing’an District, Shanghai 200002, China; 3 Advanced Dental Device Development Institute, Kyungpook National University, Daegu 41940, Korea; sonkeunbada@gmail.com; 4 Department of Dental Science, Graduate School, Kyungpook National University, Daegu 41940, Korea

**Keywords:** single crown implant, digital scan, intraoral scanner, scan body

## Abstract

The purpose of this in vitro study was to compare the accuracy of the proximal and occlusal contacts of single implant crowns fabricated with four data capture methods. The resin models were mounted on an articulator, digitized using a laboratory scanner, and saved as a standard tessellation language (STL) file to serve as the master reference model (MRM). Two different intraoral scan body (ISB) systems were evaluated: polyetheretherketone (PEEK) short scan body (SSB) and PEEK long scan body (LSB) (*n* = 12). The digital impressions (SSB and LSB) were acquired using an intraoral scanner with ISB. Two different conventional techniques were also evaluated: PEEK short scan body with coping plastic cap (CPC) and pick-up coping (PUC) (*n* = 12). The implant impressions (CPC and PUC) were recorded using a conventional impression technique. The crown and abutment were fabricated with a milling machine and then placed on the resin model and scanned using a laboratory scanner. The scanned files were saved as STL files to serve as test datasets. The MRM and test datasets were superimposed, and the mesial, distal, and occlusal distances were calculated using a 3D inspection software and statistically analyzed using the Kruskal–Wallis H test (α = 0.05). The direct data capture group had more accurate contact points on the three surfaces, with mesial contact of 64.7 (12.8) µm followed by distal contact of 65.4 (15) µm and occlusal contact of 147 (35.8) µm in the SSB group, and mesial contact of 84.9 (22.6) µm followed by distal contact of 69.5 (19.2) µm and occlusal contact of 115.9 (27.7) µm in the LSB group (*p* < 0.001). The direct data capture groups are closer to the ideal proximal and occlusal contacts for single implant crowns than the indirect data capture groups. There was no difference in the accuracy between the two types of scan body (SSB and LSB).

## 1. Introduction

Since the 1980s, dental digital workflows, including computer-aided design/computer-assisted manufacturing (CAD/CAM), digital imaging, and digital radiography, have been developed rapidly and used more increasingly for the fabrication of dental restorations [1,2,3]. As reported by several studies, one of the many advantages of the CAD/CAM method is that it presumably improves the fit and efficiency of the prosthesis compared with the conventional method (lost wax technique) [4,5]. However, an inevitable error still persists. The literature reported that the conventional workflow, which involves impression taking, production of a stone cast, and manufacture of the prosthesis, caused inaccuracies in the fabricated frameworks [6,7]. Nevertheless, the gold standard of fabricating implant restorations is a gypsum cast that is poured from a physical impression recorded with elastomeric impression material. Therefore, obtaining an accurate transfer of the implant position and angulation remains a crucial factor for achieving a satisfying prosthesis with a precise fit [8].

Digital impressions can be divided into direct data capture using direct intraoral scanning and indirect data capture using scanning of casts made from conventional impressions (impression–gypsum cast) [9]. Direct data capture using an intraoral scanner allows the data from the mouth to be sent electronically to a laboratory for the fabrication of a digital model, thus eliminating the intermediate steps involved in the conventional impression process and overcoming the errors caused by it [10,11].

Several studies have evaluated the accuracy of intraoral scanners. One study reported that digital data capture with intraoral scanners was more accurate than conventional polyether impressions [12]. Another similar study reported that direct data capture with the Lava C.O.S. intraoral scanner showed significantly higher accuracy than that of conventional impression taking and indirect data capture [9]. A study comparing the accuracy of four intraoral scanners (CS3600, Trios3, Omnicam, and True Definition) in two different situations found that CS3600 provided the best trueness and had a statistically higher mean trueness than others had [13]. However, only a few studies have evaluated the accuracy of intraoral scanners focusing on implant-supported restorations or only evaluated the data output of the digitizers.

Although a passive fit is not explicitly prescribed clinically, most people believe that high accuracy reduces the risk of long-term clinical complications [7,14]. In the absence of a buffering action from the periodontal ligament, increased occlusal loads are applied to the implant and surrounding bone, which can lead to a higher incidence of complications in implant-supported prostheses than in tooth-supported prostheses [15]. Hence, a high level of passive fit is more essential in implant-supported restorations than in tooth-supported restorations.

The digital data capture for dental implants requires an intraoral scan body (ISB). The ISB design, material, and complete scanning of the region are factors that may lead to errors [16]. With the development of dental materials, several manufacturers are offering ISB made of polyetheretherketone (PEEK) that does not need to be sprayed with non-reflective powder required for metals. Moreover, scannable healing abutments, which combine the healing abutment and ISB, have also been introduced. Consequently, one could assume that the use of scannable healing abutments made of PEEK materials would reduce dental chairside time and improve the satisfaction of patients and clinicians.

One problem that remains to be solved is the manufacture of high-quality dental crowns. A newly fabricated crown often needs to be adjusted in the dental chair for occlusal stability [17,18]. The *Journal of Prosthetic Dentistry* defined adjustment as a modification made in the dental prosthesis or natural tooth to enhance the fit, function, or patient acceptance of the prosthesis. An osseointegrated implant demonstrates extremely limited movement in the range of 10 µm, whereas a natural tooth can move up to 100 µm within its periodontal ligament. Therefore, adjustments to compensate for a certain degree of crown misfit are more important in implant-supported prostheses than in tooth-supported prostheses [19,20]. Several studies have introduced and utilized various techniques to significantly decrease the clinical time required to adjust the contacts of a newly fabricated crown and achieve optimal contact [18,21,22,23,24,25,26,27,28,29,30]. The best way to accomplish this is to either adjust the designed crown virtually or on a cast. To the best of the authors’ knowledge and a review of the current literature, no studies have measured and digitized the contacts of implant-supported restorations fabricated by CAD/CAM using four methods of digital data capture.

Thus, this in vitro study aimed to compare the accuracy of proximal and occlusal contact of single implant crown fabricated using four data capture methods. The null hypothesis was that no differences would be found in the occlusal and interproximal contacts among these four methods of data capture.

## 2. Materials and Methods

A pilot experiment was performed three times to determine the appropriate sample size. For each digital data capture method, a sample size of 12 was calculated by using a power analysis software (G*Power v3.1.9.2; Heinrich Heine University, Düsseldorf, Germany; *n* = 12 per group, actual power = 99.7%, power = 99%, α = 0.05).

**Figure 1:**
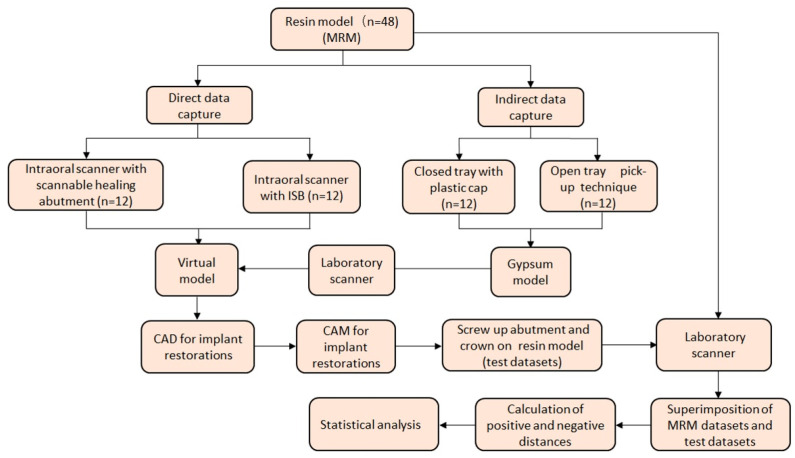
Experimental design. MRM: master reference model; ISB: intraoral scan body; CAD/CAM: computer-aided design/computer-assisted manufacturing.

Figure 1 depicts the experimental design. In the present study, two scan files were prepared to evaluate the accuracy of the contact. For the reference model, a scan file of the resin model was prepared where the implant was placed and mounted on the articulator. For the test model, implant single-unit prosthesis and customized abutment are fabricated according to the protocol of each group, and in order not to interfere with the proximal and occlusal surfaces during the bonding process of the single-unit prosthesis and the customized abutment in the resin model, the proximal and occlusal surfaces were removed and a scan file after bonding was prepared. The accuracy of the contact was evaluated by overlapping the reference model and the test model.

**Figure 2:**
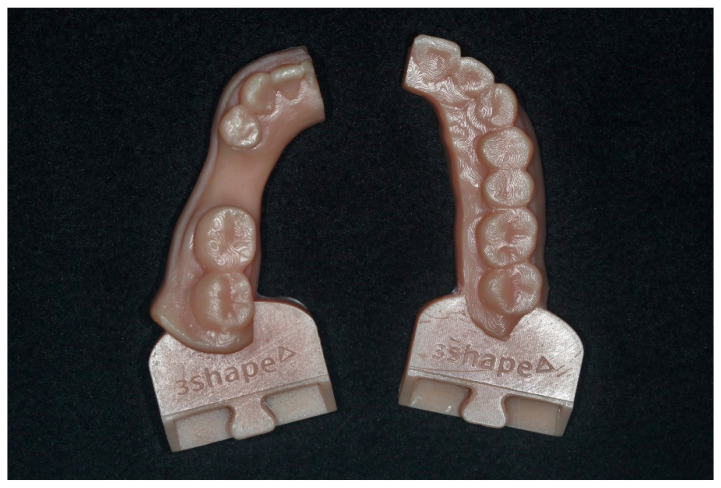
Mandibular (**left**) and maxillary (**right**) resin model.

Forty-eight customized mandibular resin models fabricated with a three-dimensional (3D) printing machine (ZENITH U; Dentis, Daegu, Korea) (Figure 2). A single bone level conical implant with 4.0 × 10 mm dimensions (TSIII SA; Osstem, Seoul, Korea) was placed in the mandibular left second premolar area using an implant surgical handpiece system (SIP20/CRB46LN; SAESHIN, Daegu, Korea). The resin models were mounted on an articulator (3Shape Articulator; 3Shape, Copenhagen, Denmark) to simulate the patient position in a clinical situation. All resin models were scanned before impressions using a laboratory scanner (E1 scanner; 3Shape, Copenhagen, Denmark), and the digitized models including the digital opposing arch and bite registrations were saved as a standard tessellation language (STL) file to serve as the master reference model (MRM). Since the models were fabricated with resin, an antireflective powder spray was not applied before scanning.

**Figure 3:**
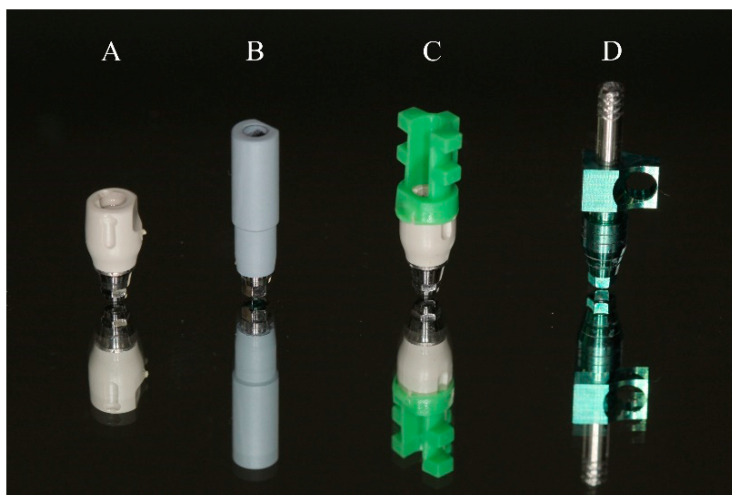
(**A**) PEEK short scan body; (**B**) PEEK long scan body; (**C**) PEEK short scan body with coping plastic cap; (**D**) pick-up coping.

Two different ISB systems were evaluated (*n* = 12): the PEEK short scan body (SSB) group (length: 7 mm) (Link type M scan body; MYFIT, Daegu, Korea) and the PEEK long scan body (LSB) group (length: 15 mm) (Osstem Regular TS scan body; Osstem, Seoul, Korea) (Figure 3A,B). Two different conventional impression techniques were also evaluated (*n* = 12): the PEEK short scan body with coping plastic cap (CPC) group (Link type M scan body) and the pick-up coping (PUC) group (hex type; Osstem, Seoul, South Korea) (Figure 3C,D).

For the conventional impressions, the operator selected the best-fit plastic tray and applied an adhesive. Using a closed tray impression technique and elastomeric impression materials (Aquasil Ultra Monophase/XLV; Dentsply Sirona, York, PA, USA), 12 conventional snap-fit implant impressions of PEEK SSB with CPC were recorded. Using an open tray impression technique with the same impression materials, 12 conventional pick-up implant impressions of PEEK SSB with PUC were recorded. Impressions of the opposing arch were also recorded with the same impression materials (Aquasil Ultra Monophase). All impression materials were handled according to the manufacturer’s recommendations; the digital impressions were recorded using an intraoral scanner (CS3600; Carestream Dental, Atlanta, GA, USA). In this study, the implant ISBs that were lower than the occlusal plane were defined as short, whereas those higher than the occlusal plane were defined as long. Twelve long ISB and 12 short ISB were utilized to digitally transfer the implant positions. Twenty-four STL files of digital scan data including the opposing arch and bite registrations recorded with the digital impression technique were exported. The sequence of scanning was performed according to the manufacturer’s guidelines. Twenty-four STL files of direct digital impression and 24 conventional impressions were sent to the laboratory for the fabrication of the custom abutments and zirconia crowns. Forty-eight implant superstructures were designed and customized by an experienced dental technician, and 48 custom implant superstructures of a single design were requested. The conventional impression was poured with type IV gypsum (GC Fujirock EP OptiXscan; GC America, Alsip, IL, USA) according to the manufacturer’s instructions. The conventional casts were stored at a room temperature of 21°C for at least 48 h until the expansion of gypsum was complete. Then, after fastening the laboratory scan body (Osstem scan body; Osstem, Seoul, Korea) to gypsum casts, all gypsum casts were scanned using a laboratory scanner (E1 scanner), and the digitized models including the digital opposing arch and bite registrations were saved as 24 STL files to serve as the indirect data capture group. All implant superstructures were designed using default settings for zirconia crowns milled from high-translucency zirconia blocks (Zeus; BIODEN, Seoul, Korea) using a 5-axis milling machine (Coritec 250i; imes-icore GmbH, Eiterfeld, Germany), and customized abutments were milled from titanium using the same milling machine and then sintered according to the manufacturer’s specifications.

**Figure 4:**
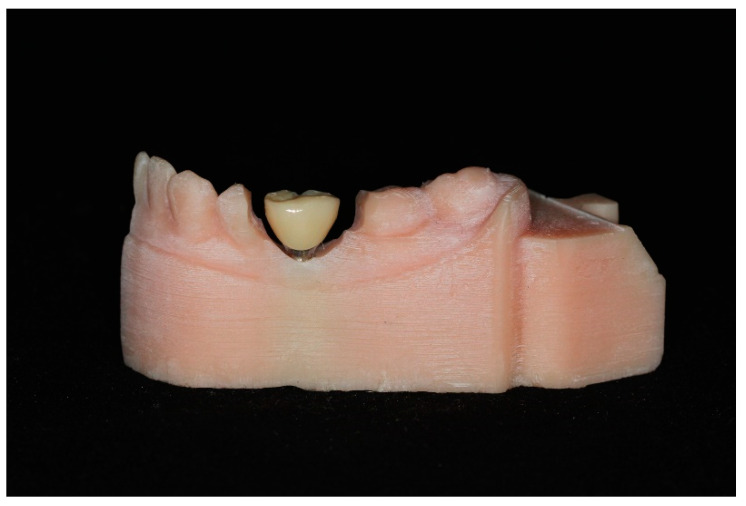
Screw-up abutment and crown on milled resin model.

The adjacent teeth were sectioned along the long axis in a buccal–lingual direction at a low-speed with a tungsten–carbide bur. The implant fixture was sealed with an adhesive tape to prevent debris from entering while grinding the adjacent teeth. The abutments were then screwed using the dental torque driver (ISD900; NSK, Tokyo, Japan) at 35 Ncm and tightened again 10 min later. Once the abutment was completely secured into the implant, the crown was secured with dual-polymerizing resin cement (RelyX U200 Clicker; 3M ESPE, St. Paul, MN, USA) on the corresponding abutment (Figure 4). All cementation procedures were performed by one operator according to the manufacturer with the corresponding crown–abutment unit then placed on the resin model and scanned using a laboratory scanner (E1 scanner).

**Figure 5:**
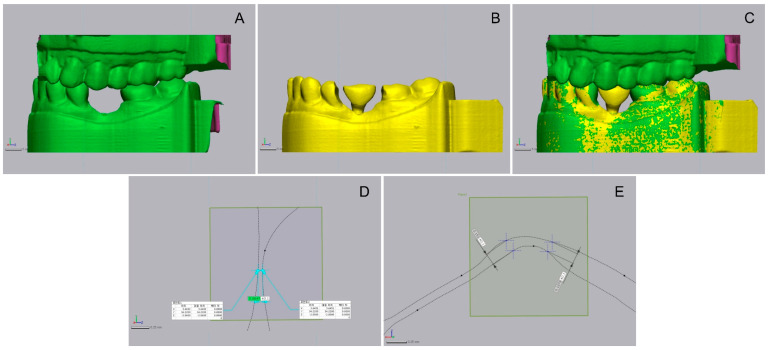
Procedure for superimposing scan data and measuring distance of contacts. (**A**) Scan data of the resin model without milling; (**B**) scan data of milled resin model with crown; (**C**) superimposition; (**D**) measurement of distance of proximal contact; (**E**) measurement of distance of occlusal contact.

All STL datasets (MRM, SSB 1–12, LSB 1–12, CPC 1–12 and PUC 1–12) were imported into the 3D inspection software program (Geomagic control X; 3D systems, Rock Hill, SC, USA). The datasets were reduced to the field of interest to minimize the errors caused by superimposition (Figure 5). Thus, all artifacts and irrelevant areas were eliminated. Each of the 12 test datasets from SSB, LSB, CPC, and PUC were aligned with the MRM dataset using the best-fit algorithm (Figure 5C). 

The software program (Geomagic control X) calculated the mesial, distal, and occlusal distances between each test and MRM dataset (Figure 5D). It also provided the Euclidean distances for each single measurement point, which can have positive or negative values in relation to the MRM dataset, and were used for data analysis [30]. The distances between a test and the reference dataset were expressed as accuracy. When there is contact and exceeding after superimposition, it is defined as a positive distance; the longest distance is calculated. When there is no contact, it is defined as a negative distance; the shortest distance is calculated. In this study, the absolute value of positive or negative distance close to 0 is defined as more accurate and vice versa. For all datasets from a single group (SSB, LSB, CPC, and PUC), the median and interquartile range were calculated to compare the accuracy of the implant-supported single crown to each other. The 3D differences were calculated and displayed in a color-coded manner using the software. For further evaluations and statistical analysis, the distances between the MRM and test datasets of each single measurement point were exported.

All data were analyzed using the statistical software (statistical package for the social sciences version 25.0; IBM, Chicago, IL, USA) (α = 0.05). First, the non-normal distribution of data was investigated using the Shapiro–Wilk test. Therefore, the result values were expressed as median and interquartile range. The Kruskal–Wallis H test was conducted to determine the differences according to the type of scan methods; as a post-test, the differences among the groups were analyzed using the pairwise comparison test.

## 3. Results

**Figure 6:**
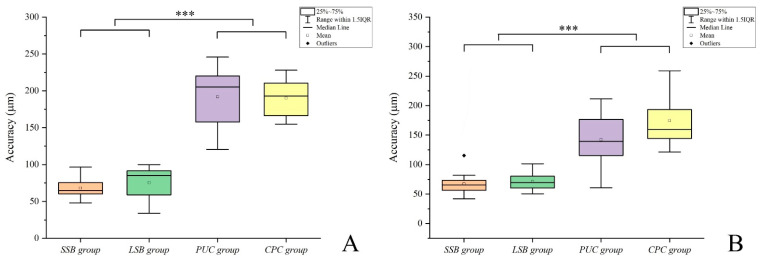
Comparison of accuracy of proximal contact among four groups. (**A**) Mesial contact of crowns; (**B**) distal contact of crowns. SSB, short scan body; LSB, long scan body; CPC, coping plastic cap; PUC, pick-up coping. ***, *p* < 0.001.

**Figure 7:**
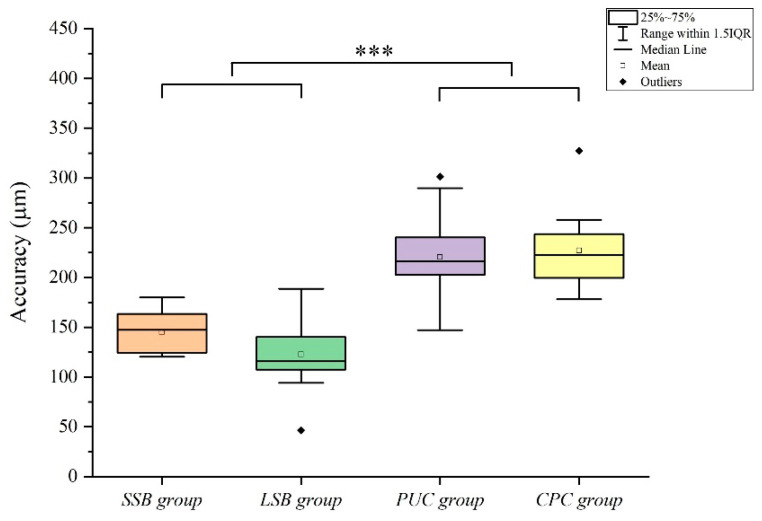
Comparison of accuracy of occlusal contact among four groups. SSB, short scan body; LSB, long scan body; CPC, coping plastic cap; PUC, pick-up coping. ***, *p* < 0.001.

Table 1 and Figure 6 and Figure 7 summarize and illustrate the results, respectively. Significant differences were found in the implant-supported single crowns among the four groups (*p* < 0.001; Table 1), which were calculated by summing up the absolute positive and negative deviations and dividing the result by the number of measured points. Here as well, the direct data capture with IOS group the more accurate contact points on the 3 surfaces: mesial contact (64.7 (12.8) µm), distal contact (65.4 (15) µm), and occlusal contact (147 (35.8) µm) in the SSB group, mesial contact (84.9 (22.6) µm), distal contact (69.5 (19.2) µm), and occlusal contact (115.9 (27.7) µm) in the LSB group (*p* < 0.001; Figure 6 and Figure 7).

The results indicated that there were no significant differences in the occlusal and proximal contacts between the direct data capture groups (SSB and LSB) (*p* = 0.964; Table 2), whereas the discrepancies in the proximal contact were significantly smaller than in the occlusal contact for the direct data capture groups (*p* < 0.001; Table 1).

## 4. Discussion

To the authors’ knowledge, this was the first to compare different implant impression techniques to evaluate the contacts for seating single crown implants. The null hypothesis that no differences would be found in the occlusal and interproximal contact among these four methods of data capture was rejected (*p* < 0.001). In addition, the accuracy of proximal contact and accuracy of occlusal contact was significantly different (*p* < 0.05). In the groups using scan bodies (SSB and LSB), the accuracy of the occlusal contact was significantly increased compared to the proximal contact (*p* < 0.05), but the conventional methods (CPC and PUC) showed that the occlusal contact was no significant compared to the proximal contact (mesial or distal contacts). Therefore, due to the increase in the accuracy value of the significant occlusal contact of the groups using the scan body (SSB and LSB), the deviation among the conventional methods (CPC and PUC) and the groups using the scan body (SSB and LSB) was reduced in the comparison of occlusal contact than in the comparison of proximal contact.

Figure 6 shows the accuracy of the proximal contact, and Figure 7 shows the accuracy of the occlusal contact. There is less difference among the four groups in the accuracy of the occlusal contact than in proximal contact. This is because the error of the occlusal contact of the groups (SSB and LSB) using the scan body increased (Table 1). In previous studies, the vertical displacement of the scan body was reported in the process of tightening the scan body to the implant [31,32]. It has also been reported that the vertical displacement of the scan body can affect the vertical position of the final prosthesis [31,32].

This study aimed to evaluate the entire manufacturing process from four different digital data capture methods, instead of only evaluating the accuracy of implant digital capture. Several previous studies have already superimposed the test and reference datasets using a repeated best-fit algorithm to examine the trueness of complex objects and surfaces [9,23]. These studies found that the choice of best-fit alignment, reference scanner, and the digitization method can affect the accuracy regardless of the operators. In this study, one reference scanner (E1 scanner) with an accuracy of 10 µm was used to create the 3D datasets, and the measuring software (Geomagic control X) was then utilized to calculate the arithmetic value from positive and negative distances.

The approach applied in the present study used the absolute positive and negative values to estimate the contacts of each test dataset in relation to the reference. One median value for each group was calculated from these values. However, the values are separated into positive and negative ranges. Therefore, for the average distance between the test and reference datasets, the average of absolute values of the Euclidean distances was calculated for each group. This absolute value for each group was interpreted in the present study as the accurate value of the occlusal contact, mesial contact, and distal contact, respectively. The direct digitalization groups showed better accuracy than did the indirect digitalization groups, and all contact of the crown was no significant difference in the direct digitalization groups. This indicates that (1) the digital model obtained via the direct method with ISO is more accurate than that obtained via the indirect method with elastic materials and laboratory scanner and (2) the type of scan body has no effect on the accuracy of the single implant crown [16].

The digital data capture and matching operation could affect the accuracy of the image matching process [29]. Therefore, the data obtained in this test are also certified. When an ISB is used for single implant crowns, the height and shape of the ISB do not affect the final crown. From a clinical perspective, the use of SSB (scannable healing abutment) is more recommended because no disassembly is required before the final crown is restored, which can shorten the clinical time and increase the comfort of patients.

In the present study, differences in the four methods of digital data capture are methods of directly tightening the scan body to the implant fixture and scanning it with an intraoral scanner (SSB and LSB groups), and methods of making a working cast using the conventional impression method (elastomeric impression) and scanning it with a desktop scanner (CPC and PUC groups). In this experiment, four different methods were used to obtain the virtual models, which were used to fabricate the implant prostheses. Although the trial design attempted to simulate some of the clinical conditions during this process, conditions such as saliva, blood, and soft tissue could not be simulated. The present study unified the operation, design, and manufacturing processes besides data acquisition. Especially, the MRM and test datasets obtained using the same scanner were superimposed to avoid a greater error [28].

Although all digital impressions were recorded by one skilled operator (X.R.), it may be assumed that the reproducibility would decrease with an increase in the operator the values are separated of the process remained the same. The higher inaccuracy of the CAD/CAM based on the elastic impression materials, gypsum master cast, and desktop scanner digitalization can be explained by the numerous potential sources of errors during the long procedure until a construction dataset can be obtained [24,25]. The scanning software could filter out the possible outliers and smoothen the surface of the construction datasets. This suggests that all of the factors could influence the precision of the single datasets captured.

The superimposition process can also influence the measurement procedure and the results. As determined by the pilot study, the maximum allowable alignment error and/or deviation had a positive average of 2 µm or less and a root mean square estimate of 4 µm or less for each specimen analyzed. If any specimens had a higher error, they were realigned until a smaller deviation was obtained. Therefore, the measurement errors caused by superimposition can be eliminated. Additionally, the accuracy of the desktop scanner used for the evaluation of the present study must be considered. The accuracy of the desktop scanner used in the present study was calibrated before the experiment, and the scanning accuracy of less than 10 µm was verified by the manufacturer. The present study evaluated the contact accuracy of the implant prosthesis manufactured by each group, but the previous study evaluated the strain around implant [33]. The implant prosthesis should be passively connected to the implant for a good long-term prognosis, so that less strain around implants should be given [33]. The reason for evaluating the contact accuracy of the implant prosthesis in the present study is that the contact accuracy can affect the strain around implant. Therefore, further studies are needed on the effect of the contact accuracy of the implant prosthesis on the strain around implant.

Forty-eight models of missing mandibular left second premolars were 3D printed, and implant fixtures were placed in the same position using a surgical guide. Although the implants were placed by an experienced clinician, they were found to be in different positions after placement with the largest deviation of 7.4 degrees and 2.4 mm depth. Beatriz Gimdept et al. showed that the angulation and depth of the implant itself do not significantly influence the accuracy of the digital impression [26].

Another factor of the implant workflow that may cause small deviations in the accuracy is the matching of the virtual scan body from the CAD software. It depends on the scan data quality and the surface matching algorithm of the software. The poor quality of scanned datasets may result in an incorrect matching of the scan body and an error in the analog position in the virtual model. Stimmelmayr et al. reported an average discrepancy of 39 µm in the fit of the ISB on the original implants and only 11 µm on the implant analogues [27]. A half arch model and commercial implant fixtures were used in the present study to simulate the clinical conditions and to improve the scanning quality.

In the present study, factors influencing the accuracy of proximal and occlusal contacts in the digitalization impression procedure differ in the four groups. There is a marked difference between the groups (SSB and LSB) that perform a digitalization impression with an intraoral scanner by directly tightening the scan body in the oral cavity, and the groups (CPC and PUC) that produce a working cast through a physical impression procedure and perform a digitalization impression with a desktop scanner. The SSB and LSB groups are the most influencing the accuracy of proximal and occlusal contacts, the accuracy of the intraoral scanner, and the CPC and PUC groups, the process of making a working cast through a physical impression procedure and the accuracy of a desktop scanner. These differences should be verified through further studies.

These in vitro results indicate that anatomic zirconia single crowns made by the CAD/CAM method from the direct data capture technology can offer better results than those from the indirect methods. However, the impression procedure and digitalization can be influenced by a variety of factors in the oral cavity. Thus, further in vivo studies are required to confirm the trueness of these findings in clinical practice. Moreover, visualizing the magnified images of the crown’s contacts on the screen might help dentists to effectively improve the process of adjustments and impressions in the future.

The accuracy of the intraoral scanner may vary depending on the intraoral conditions [34], and the type of intraoral scanner also affects accuracy [35,36]. In the present study, one intraoral scanner (CS3600) was used, and the evaluation was conducted. Additional studies using various intraoral scanners are needed. In addition, from the perspective of the present study, in a previous study, biotechnologies to analyze oral tissues were evaluated as novel approaches through bioimpedance examination [37]. Since oral tissues play an important role in the long-term prognosis of implant prostheses, additional studies through bioimpedance examination should be performed.

There are several limitations in the present study. The present study shows a larger space than the area of the missing premolar, and the crown emergence profile shows a convex shape (Figure 2 and Figure 4). For this reason, the present study did not show standardized casts, because the working casts in an actual patient with a missing premolar were used. In addition, the reason why the crown emergence profile has a convex shape is that the implant was placed on the working cast before the emergence profile of the gingiva was formed after implantation. In addition, there is a limitation of the inability to restore the mobility of adjacent and opposite arch teeth. Finally, the present study fabricated an implant prosthesis with a working cast of the half arch. A working cast of the half arch is often used for single-unit implant prosthesis in clinical practice, but there is room for deviation in clinical experimental conditions. Therefore, further studies should be conducted, taking into account these limitations.

## 5. Conclusions

The direct data capture groups are closer to the ideal proximal and occlusal contact of the single implant crown fabricated using an intraoral scanner than the indirect data capture groups. Since there was no difference in the accuracy of proximal and occlusal contacts of the single implant crown fabricated by two types of scan body with different lengths, the length of the scan body did not affect the accuracy of the contact. The accuracy of the occlusal contact was lower than the proximal contact in the direct data capture with the IOS group. Therefore, the clinician should be more careful about the error of occlusal contact when fabricating a single implant crown using an intraoral scanner.

## Figures and Tables

**Table 1:** Comparison of 3D displacements according to method of data capture.

Contact Type	SSB	LSB	CPC	PUC	*p*
	Median (Interquartile Range), µm				
Mesial contact	64.7 (12.8) ^Aa^	84.9 (22.6) ^Aa^	192.9 (40.5) ^Bab^	205.1 (54.1) ^Bab^	<0.001 *
Distal contact	65.4 (15.0) ^Aa^	69.5 (19.2) ^Aa^	159.6 (41.6) ^Ba^	139.4 (48.1) ^Ba^	<0.001 *
Occlusal contact	147.5 (35.8) ^Ab^	115.9 (27.7) ^Ab^	222.5 (38.5) ^Bb^	216.3 (30.8) ^Bb^	<0.001 *
*p*	<0.001 *	<0.001 *	0.009 *	0.002 *	

Same uppercase letters in same row and same lowercase letters in same column show no statistical significance (*p* > 0.05). SSB, short scan body; LSB, long scan body; CPC, coping plastic cap; PUC, pick-up coping. * Significance determined by Kruskal–Wallis H test, *p* < 0.05.

**Table 2:** Results of factorial ANOVA of ranks.

Source of Variation	*p*
Impression technique type	<0.001 *
Scan body type	0.964
Impression technique × proximal and occlusal contact	<0.001 *

* Significance determined by factorial ANOVA of ranks, *p* < 0.05.

## Data Availability

Data is contained within the article.
